# Undifferentiated pleomorphic sarcoma of the retroperitoneum mimicking a cortisol‐ and catecholamine‐secreting adrenal tumor

**DOI:** 10.1002/iju5.12436

**Published:** 2022-03-22

**Authors:** Takayoshi Fuu, Akihiro Yano, Shinji Urakami

**Affiliations:** ^1^ Department of Urology Toranomon Hospital Tokyo Japan

**Keywords:** catecholamine, cortisol, mimicking tumor, retroperitoneal tumor, undifferentiated pleomorphic sarcoma

## Abstract

**Introduction:**

Retroperitoneal tumors with endocrine abnormalities are suspected to be functional adrenal tumors. Retroperitoneal soft tissue sarcomas are rare tumors, without endocrine potential.

**Case presentation:**

A 60‐year‐old male was referred for a 15 cm mass in the left suprarenal space. His plasma cortisol and catecholamine levels were elevated. He underwent open left adrenalectomy with radical nephrectomy and his endocrinological abnormalities were improved. Pathological findings suggested that it had originated from the retroperitoneal fat tissue, and a diagnosis of undifferentiated pleomorphic sarcoma was made based on the results of immunohistochemical analysis and fluorescence in situ hybridization. Interestingly, neither cortisol nor catecholamine was elevated when, 6 months after surgery, local recurrence developed.

**Conclusion:**

This is the first reported case of undifferentiated pleomorphic sarcoma accompanied by high levels of cortisol and catecholamine. We should keep in mind the possibility of tumors like retroperitoneal soft tissue sarcomas inducing endocrine abnormalities.

**Abbreviations**
DST: dexamethasone suppression test
MIBG: ^123^I‐metaiodobenzylguanidine
RPS: retroperitoneal soft tissue sarcoma
UPS: undifferentiated pleomorphic sarcoma

**Keynote message**

Retroperitoneal tumors with endocrine abnormalities are normally suspected to be functional adrenal tumors. There are unique cases of other tumors like sarcomas mimicking cortisol‐ or catecholamine‐secreting adrenal tumors. We should keep in mind the possibility of non‐adrenal tumors inducing endocrine abnormalities.

## Introduction

Retroperitoneal masses around adrenal lesion with endocrine abnormalities are suspected to be functional adrenal tumors, which may be malignant or benign. A differential diagnosis is adrenal cortex adenoma or carcinoma with oversecreting of cortisol, or pheochromocytoma with oversecreting of catecholamine.^1^ Initial diagnosis includes radiological phenotypical evaluation and biochemical assessment of tumor hormonal activity.^2^ Most advocate resection of a mass larger than 4 cm if the patient is a surgical candidate, unless there is clearly a transient, infectious, or benign cause.^1^

RPSs are rare tumors that account for approximately 12–15% of all soft tissue sarcomas with an incidence rate of 2.7 per million.^3^ RPSs are frequently incidental findings in the work‐up for non‐related diseases and can grow to an extremely large size in the retroperitoneum before symptoms of abdominal pain, back pain, bowel obstruction or a palpable abdominal mass.^4^ Surgical resection is the only hope for a cure and is therefore the treatment of choice for localized disease.^4^ Here we report a unique case of suprarenal RPS mimicking a cortisol‐ and catecholamine‐secreting adrenal tumor.

## Case presentation

**Fig. 1:**
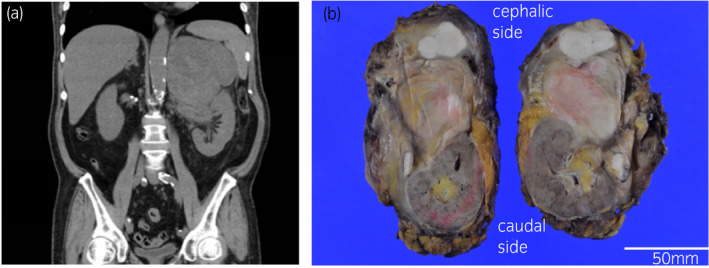
(a) Computed tomography of sagittal abdomen on admission (b) Macro image of the whole resected specimen. The tumor is on the cephalic side and the left kidney is on the caudal side.

A 60‐year‐old male presented with malaise and 10 kg weight loss over a period of 1 year. He also had a fever of over 38° and tachycardia. He had a history of hypertension, but no transient elevation of blood pressure, headache or profuse sweating. Blood tests revealed inflammatory findings (C reactive protein 15.8 mg/dL), anemia (hemoglobin 8.3 g/dL) and deterioration of glucose tolerance (HbA1c 7.5%) (Table 1). Computed tomography showed a heterogeneous mass 15 cm in size in the left suprarenal space (Fig. 1a). Magnetic resonance imaging scan showed an internal heterogeneous mass with mixed high and low signal on T2‐weighted images and fluorodeoxyglucose‐positron emission tomography showed hyperintensity in the mass. For further assessment, detailed blood and urine tests were performed, the results of which are shown in Table 2. Briefly, serum cortisol levels were elevated at midnight and were not suppressed by 1 mg dexamethasone. Plasma adrenocorticotropic hormone level was markedly suppressed. In addition, 24‐h urinary noradrenaline and normetanephrine levels were greater than three times the normal upper limit. These results indicated that the suprarenal tumor was an adrenal tumor, secreting both cortisol and catecholamine, although MIBG‐scintigraphy showed no uptake in the tumor. For more definitive diagnosis and treatment, we performed open left adrenalectomy with radical nephrectomy, with blood transfusions and 12 mg of oral doxazosin mesylate as preoperative preparation. No intraoperative hypertension was observed.

**Table 1:** Laboratory findings on admission

Hematology			Biochemistry		
White blood cell	12 900	/μL	Total protein	7.6	g/dL
Neutrophil	77.2	%	Albumin	2.3	g/dL
Eosinophil	0.3	%	Blood urea nitrogen	9	mg/dL
Monocyte	7.2	%	Creatinine	0.53	mg/dL
Lymphocyte	14.7	%	Sodium	133	mEq/L
Red blood cell	3.78	×10^6^/μL	Potassium	4.4	mEq/L
Hemoglobin	8.3	g/dL	Chloride	97	mEq/L
Platelet	769	×10^3^/μL	Calcium	8.6	mg/dL
Coagulation			Total bilirubin	0.4	mg/dL
Prothrombin time	69.7	sec	Aspartate aminotransferase	46	IU/L
Activated partial thromboplastin time	29.8	sec	Alanine aminotransferase	53	IU/L
D dimer	0.4	μg/mL	Alkaline phosphatase	1299	IU/L
			γ glutamyl transpeptidase	252	IU/L
			Lactate dehydrogenase	226	IU/L
			HbA1c	7.5	%
			C reactive protein	15.8	mg/dL
			Ferritin	954	μg/dL
			Iron	12	μg/L
			Tumor necrosis factor α	2.19	pg/mL
			Interleukin 6	97.9	ng/L

**Table 2:** Endocrinological data on admission

Endocrinology	
	Value unit (reference value)
24 h urince catecholamine	984 μg/day (34–198)
24 h urince dopamin	2730 μg/day (280–1100)
24 h urince noradrenaline	960 μg/day (31–160)
24 h urince metanephrine	1.12 mg/day (0.14–0.46)
24 h urince normetanephrine	0.97 mg/day (0.10–0.28)
24 h urince vanillylmandelic acid	5.47 mg/day (1.50–4.90)
Plasma adrenocorticotropin hormone	3.7 pg/mL (7.2–63.3)
Plasma cortizol	21.4 μg/dL (4.5–21.1)
Plasma adrenocorticotropin hormone (DST 1 mg)	<1.0 pg/mL (7.2–63.3)
Plasma cortizol (DST 1 mg)	7.7 μg/dL (4.5–21.1)
Plasma dehydroepiandrosterone sulphate	1022 ng/mL (270–1400)
Plasma testosterone	50.4 ng/dL (142.4–923.1)
Plasma aldosterone	6.4 ng/dL (3.0–15.9)
Plasma renin activity	18.5 ng/mL/h (0.2–2.3)

**Fig. 2:**
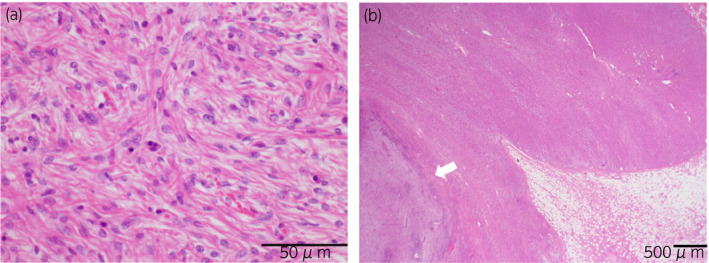
(a) Diffuse proliferation of spindle and multi‐forming cells with large hyperchromatic nuclei and narrow eosinophilic cytoplasm on hematoxylin and eosin‐stained sections (original magnification × 400) (b) Image of left adrenal gland and infiltrating tumor (white arrow) on hematoxylin and eosin‐stained sections (original magnification × 20).

The tumor was a tan‐colored, multilobulated solid mass 30 × 25 × 13 cm in size, situated in the retroperitoneum around the superior pole of kidney, and it extended to the hilum of the left kidney (Fig. 1b). Histology revealed atypical spindle cells with a fascicular architecture intermingled with lymphoplasmacytic infiltrates (Fig. 2a). On immunohistochemistry, the tumor cells were positive for CD34 and negative for AE1/3, EMA, desmin, SMA, S100, c‐KIT, SF‐1, chromogranin A, synaptophysin, MDM2, and CDK4. The Ki67 labelling index was 12%. Fluorescence in situ hybridization revealed no amplification of MDM2. A definitive diagnosis of UPS was made. The left adrenal gland was slightly invaded, and hyperplasia was observed in the adrenal cortex but not in the adrenal medulla on hematoxylin and eosin‐stained sections (Fig. 2b).

The patient was discharged 10 uneventful days following surgery, after recovering from fever and tachycardia. Catecholamine and cortisol levels had been normalized prior to discharge. Unfortunately, the patient developed local recurrence 6 months after the operation. Intriguingly, neither catecholamine nor cortisol levels were elevated at recurrence. The patient received radiotherapy to the local recurrent site with a total dose of 60 Gy in 25 fractions, and has since remained in stable condition without distant metastasis for a period of 5 months since the end of the radiotherapy.

## Discussion

In the present case, suprarenal RPS mimicked a cortisol‐ and catecholamine‐secreting adrenal tumor. To our knowledge, this is the first reported case of UPS accompanied by high levels of cortisol and catecholamine. UPS, formerly called malignant fibrous histiocytoma and declassified by the World Health Organization in 2002, is a rare and malignant subtype characterized by a lack of specific immunohistochemical markers for any specific lineage of differentiation.^5^, ^6^ It represents the fourth most common soft tissue sarcoma and has an incidence of about 0.08–1 per 100 000.^7^ In the present case, both preoperative imaging modalities and hormonal studies indicated a possible functional adrenal tumor secreting cortisol and catecholamine. Many case reports have described adrenal tumors, including malignant ones, that over‐secrete these two hormones.^8^, ^9^ In this case, however, pathological examination revealed that the tumor was UPS, without potential of producing the hormones. In addition, neither catecholamine nor cortisol levels were elevated when the local recurrence occurred. This implies that the tumor itself had not caused the elevations in these hormones that were seen when the patient first presented. One possible explanation for the high preoperative cortisol levels is adrenal cortex hyperplasia. Some soft tissue sarcomas have been reported to cause paraneoplastic syndrome, in which the tumor‐derived growth factors and cytokines produced by these tumors induce adrenal cortex hyperplasia, which causes the adrenal gland to over‐secrete cortisol.^10^, ^11^, ^12^ Some previous reports have described retroperitoneum tumors that mimicked pheochromocytoma.^13^, ^14^, ^15^, ^16^, ^17^ One hypothesis proposed by Ajmi *et al*. is that paracrine stimulation of adrenal secretion by the RPS results in elevated catecholamine.^13^ In the present case, however, imaging studies including MIBG scintigraphy did not show the presence of any neuroendocrine tumors such as paraganglioma. An alternative explanation is that catecholamine was overproduced due to compression of the renal vessels or the surrounding nerves by the large tumor. The former hypothesis has been proposed previously in connection with several other cases, indicating that the over‐secretion of catecholamine is triggered by the renin‐catecholamine pathway.^13^, ^14^ The latter hypothesis is based on the anatomical observation of the sympathetic nerves surrounding the renal vessels.^18^

## Conclusion

We presented a unique case of suprarenal UPS mimicking a cortisol‐ and catecholamine‐secreting adrenal tumor. Even when we strongly suspect functional adrenal tumors, we should keep in mind the possibility of other tumors like RPS inducing endocrine abnormalities.

## Author contributions

Takayoshi Fuu: Data curation; Writing – original draft. Akihiro Yano: Writing – review & editing. Shinji Urakami: Supervision.

## Conflict of interest

The authors declare no conflict of interest.

## Approval of the research protocol by an Institutional Reviewer Board

Not applicable.

## Informed consent

Written informed consent was obtained from the patient and can be suppled on request.

## Registry and the Registration No. of the study/trial

Not applicable.

## References

[1] Nieman LK . Approach to the patient with an adrenal incidentaloma. J. Clin. Endocrinol. Metab. 2010; 95: 4106–13. 20823463 10.1210/jc.2010-0457 PMC2936073

[2] Cyrańska‐Chyrek E , Grzymisławska M , Ruchała M . Pułapki w diagnostyce incydentaloma nadnerczy. Endokrynol. Pol. 2017; 68: 360–77. 28660993 10.5603/EP.2017.0028

[3] Bonvalot S , Rivoire M , Castaing M *et al*. Primary retroperitoneal sarcomas: a multivariate analysis of surgical factors associated with local control. J. Clin. Oncol. 2009; 27: 31–7. 19047280 10.1200/JCO.2008.18.0802

[4] Messiou C , Moskovic E , Vanel D *et al*. Primary retroperitoneal soft tissue sarcoma: imaging appearances, pitfalls and diagnostic algorithm. Eur. J. Surg. Oncol. 2017; 43: 1191–8. 28057392 10.1016/j.ejso.2016.10.032

[5] Doyle LA . Sarcoma classification: an update based on the 2013 World Health Organization classification of tumors of soft tissue and bone. Cancer 2014; 120: 1763–74. 24648013 10.1002/cncr.28657

[6] Zhu Y , Hao D , Tang X , Sun L . Undifferentiated high‐grade pleomorphic sarcoma of ethmoid sinus: a case report and literature review. Braz. J. Otorhinolaryngol. 2018; 84: 389–92. 28629789 10.1016/j.bjorl.2017.05.004 PMC9449229

[7] Demetri GD , Antonia S , Benjamin RS *et al*. Soft tissue sarcoma. J. Natl. Compr. Canc. Netw. 2010; 8: 630–74. 20581298 10.6004/jnccn.2010.0049

[8] Alexandraki KI , Michail OP , Nonni A *et al*. Corticomedullary mixed adrenal tumor: case report and literature review. Endocr. J. 2009; 56: 817–24. 19461165 10.1507/endocrj.k09e-010

[9] Alsabek MB , Alhmaidi R , Ghazzawi B , Hamed G , Alseoudi A . Mixed corticomedullary adrenal carcinoma – case report: comparison in features, treatment and prognosis with the other two reported cases. Int. J. Surg. Case Rep. 2017; 31: 254–61. 28199934 10.1016/j.ijscr.2017.01.010 PMC5310178

[10] Dorn C , Bugl S , Malenke E *et al*. Paraneoplastic granulocyte colony‐stimulating factor secretion in soft tissue sarcoma mimicking myeloproliferative neoplasia: a case report. BMC Res. Notes 2014; 7: 313. 24885681 10.1186/1756-0500-7-313 PMC4039653

[11] Des Guetz G , Mariani P , Freneaux P , Pouillart P . Paraneoplastic syndromes in cancer. J. Clin. Oncol. 2004; 22: 2242–3. 15169814 10.1200/JCO.2004.08.019

[12] Angelousi A , Kyriakopoulos G , Nasiri‐Ansari N , Karageorgou M , Kassi E . The role of epithelial growth factors and insulin growth factors in the adrenal neoplasms. Ann. Transl. Med. 2018; 6: 253. 30069455 10.21037/atm.2018.05.52 PMC6046285

[13] Trimeche Ajmi S , Marmouch H , Trabelsi A *et al*. Retroperitonial liposarcoma mimicking pheochromocytoma. Pathologica 2008; 100: 470–2. 19475889

[14] Pekic S , Damjanovic S , Djurovic M *et al*. Retroperitoneal malignant fibrous histiocytoma mimicking pheochromocytoma. Endocrine 2004; 24: 099–104. 10.1385/ENDO:24:1:099 15249709

[15] Kiriakopoulos A , Papaioannou D , Linos D . Adrenal cortical oncocytoma mimicking pheochromocytoma. Hormones 2011; 10: 76–9. 21349810 10.14310/horm.2002.1296

[16] Caliskan S , Yencilek E . Large B‐cell lymphoma mimicking adrenal pheochromocytoma. Indian J. Med. Res. 2013; 138: 276. 24056610 PMC3788220

[17] Lu J , Xiong XZ , Li L , Cheng NS . An unusual retroperitoneal tumour mimicking adrenal pheochromocytoma. Dig. Liver Dis. 2015; 47: e18. 26210307 10.1016/j.dld.2015.06.009

[18] García‐Touchard A , Sañudo JR . Renal denervation. Importance of knowledge of sympathetic nervous system anatomy in refining the technique. Rev. Esp. Cardiol. 2019; 72: 531–4. 31097344 10.1016/j.rec.2019.01.016

